# Take A Breath: study protocol for a randomized controlled trial of an online group intervention to reduce traumatic stress in parents of children with a life threatening illness or injury

**DOI:** 10.1186/s12888-016-0861-2

**Published:** 2016-05-27

**Authors:** Meredith Rayner, Frank Muscara, Anica Dimovski, Maria C. McCarthy, Jackie Yamada, Vicki A. Anderson, Kylie Burke, Robyn Walser, Jan M. Nicholson

**Affiliations:** Clinical Sciences, Murdoch Childrens Research Institute, The Royal Children’s Hospital, Flemington Road, Parkville, Victoria 3052 Australia; Children’s Cancer Centre, The Royal Children’s Hospital, Flemington Road, Parkville, Victoria 3052 Australia; Parenting and Family Support Centre, School of Psychology, The University of Queensland, Brisbane, Queensland 4072 Australia; University of California, Berkeley and TL Consultation Services, Menlo Park, California USA; Judith Lumley Centre, La Trobe University, 215 Franklin St, Melbourne, Victoria 3000 Australia

**Keywords:** Parents, Pediatric illness, Post-traumatic stress, Randomized controlled trial, Acceptance and commitment therapy, Online group intervention

## Abstract

**Background:**

A substantial proportion of parents whose child is diagnosed with a life-threatening illness, experience high levels of distress that can lead to long-term difficulties in mental health, family functioning and child adjustment. This study evaluates the efficacy of an Acceptance Commitment Therapy-based group intervention designed to reduce distress symptoms in these parents. The program is delivered using videoconferencing to overcome factors that prevent participation in traditional face-to-face therapy.

**Method/design:**

The study is a randomized control trial of the Take A Breath group intervention for parents demonstrating elevated symptoms of acute stress, delivered via videoconferencing in six 90 min group sessions. Participants are the primary caregivers of children aged 0 to 18 years admitted for a life threatening illness or injury to the Oncology, Cardiology, Neurology or Intensive Care Departments of a tertiary pediatric hospital. Parents will be randomized to intervention or waitlist control 4–10 months after their child’s diagnosis. Measures will be collected prior to and immediately post intervention for intervention and waitlist parents to assess program efficacy. Intervention parents will be followed up at 6 months to assess the maintenance of program effects. We predict that intervention parents will show fewer symptoms post intervention than waitlist parents (primary outcomes: traumatic stress, depression, anxiety, stress symptoms), reflecting improvements in the psychological skills addressed in the intervention (mediating factors). It is anticipated that reductions in mental health difficulties for intervention parents will be maintained up to 6 months post-intervention and will be associated with broader improvements in parents’ adjustment, child adjustment and child wellbeing (secondary outcomes).

**Discussion:**

This study is unique in evaluating a group intervention delivered to parents of children affected by of a diverse range life-threatening illness or injury. Online communication technology is employed to reduce participation barriers. If proven efficacious, this trans-diagnostic approach offers the potential for broad use as part of the suite of psychosocial services provided to families through tertiary pediatric settings.

**Trial registration:**

ACTRN12611000090910. Trial Registration Date: 14/09/2011

**Protocol Date/version:** September 2015, version M

**Study Status:** Ongoing

## Background

Life-threatening medical conditions affect thousands of Australian children and their families every year. Facing the possibility of their child’s death or lasting impairment, while negotiating complex medical processes and making difficult treatment decisions, is an experience that can overwhelm even the most resilient parent [1]. Many parents report enduring symptoms of trauma including intense feelings of fear, anxiety, helplessness, intrusive thoughts and hypervigilance [2]. Post-traumatic stress disorder has been reported for up to 29 % of mothers and up to 18 % of fathers 6–12 months after child diagnosis [3–6], with a further 46 % of mothers and 28 % of fathers experiencing significant sub-threshold levels of post-traumatic stress symptoms 6 months after their child’s cancer diagnosis [5]. These symptoms limit parents’ ability to manage daily activities and provide the recovering child with an optimal post-hospital environment [7]. This paper presents the study protocol of a randomized control trial (RCT) that seeks to evaluate a group psychological intervention delivered to parents in their homes, via videoconferencing. The intervention aims to improve parent mental health, thereby optimizing the child’s recovery environment.

Parental distress following child diagnosis is normative and potentially adaptive – parents of seriously ill children are generally well functioning individuals placed in extraordinary circumstances. Most adapt with time and the support of family and friends, and distress symptoms subside. However, higher levels of distress reactions in this early phase have been shown to predict later, persistent parent mental health difficulties [5, 8, 9] placing parents and the ill child at high risk of mental health problems [10, 11]. Persistent distress can impair a parent’s ability to respond to the demands of their child’s illness [12–14], and result in the utilization of more hospital resources [15]. It is not surprising that the combination of parental trauma and poorer family functioning predicts the longer-term psychological, behavioral and general wellbeing of the ill child [6, 10, 11, 16–18], and has been associated with elevated rates of child psychological, social and health difficulties, including weight and eating difficulties, behavioral, social and emotional problems and negative body image [14, 15].

The ‘Take A Breath’ (TAB) group program was developed as an early intervention for parents with persistent symptoms of distress. It was designed to provide parents with the skills to manage the psychological challenges presented by their child’s illness, with the aim of preventing more serious long-term mental health difficulties. Preliminary (non-controlled) outcomes from pilot studies of TAB delivered face-to-face and online have been described elsewhere [19, 20]. Briefly, the program utilizes an Acceptance and Commitment Therapy (ACT) approach [21], including ACT’s key elements of acceptance, mindfulness, values clarification and goal setting. These are logical treatment approaches to manage the intrusive thoughts, avoidance and high levels of emotional arousal which are among the most common distressing symptoms in parents of children with a serious illness or injury [22–24]. A group-based approach was selected for its advantages in helping to normalize the challenges faced by parents, the opportunities for providing peer-support and modelling of coping strategies, and the efficiency and cost-effectiveness of group compared to individual treatment. While the targeted participants for the program are parents who are the primary carers of the ill child (predominantly mothers), partners are also encouraged to attend, as they are a key source of support for enacting any changes [23] and may also benefit from the program themselves. The intervention is delivered 4–10 months after child diagnosis, a time when the acute management of the child’s condition has eased and parents are more able to consider their own needs [15].

Research with parents of ill children has found problematic levels of uptake and attendance for group interventions [23]. Barriers to attending face-to-face programs include recurrent illness and/or ongoing disability in the child, time, childcare and financial pressures that have arisen from the child’s illness, and a preference to spend time at home following family separations associated with the child’s hospitalization [22–24]. A recent systematic review of family adjustment to childhood cancer [14] highlighted the complex and profound effects of child illness on family life, including the child’s needs taking priority over those of parents and siblings, lack of time for ‘non-essential’ activities, extended periods of family separation during the child’s treatment, and a resulting desire to protect family time. Traditional face-to-face interventions may be difficult for many parents given these multiple demands. The current trial utilizes videoconferencing technology as the method of delivery. This allows parents to take part in the program in the comfort of their own homes and removes burden of additional travel costs, child care needs and limited time.

The use of technology to deliver interventions in the home is a promising alternative to traditional face-to-face delivery [25]. Internet based interventions that also include clinician involvement appear to be more effective [26] and maintain treatment effects for longer periods [27] than self-directed treatments. Videoconferencing is a method of electronic delivery that allows client and clinician to see and hear each other, providing the advantages of real time verbal and nonverbal interactions [28]. One-on-one videoconferencing (a single client and clinician) is comparable with face-to-face interventions in terms of effectiveness, client ratings of the therapeutic relationship, client satisfaction [25, 29] and treatment retention [30] for a variety of psychological conditions [29]. A recent systematic review found videoconferencing psychotherapy has been used in a variety of therapeutic formats, with diverse populations, and was generally associated with good user satisfaction, having similar clinical outcomes to traditional face-to-face psychotherapy [31].

Early data on videoconferencing for group therapy are promising [31]. Studies have reported equivalent outcomes to those found in face-to-face groups for 125 clients with posttraumatic stress disorder [25, 29, 30] and seven clients with traumatic brain injury [28]. King and colleagues [32] found high client acceptability and clinical effectiveness for clients with substance addictions (*N* = 37). In comparison with face-to-face therapy, videoconferencing groups for veterans with posttraumatic stress disorder (*N* = 38) showed equal levels of adherence, empathy and rapport, and more favourable ratings of therapist competence in the videoconferencing conditions [33]. Our uncontrolled feasibility study shows that TAB delivered online (*N* = 13) was associated with significant reductions on three measures of parental psychological symptoms from pre to 6 month follow-up [20].

### Aims and hypotheses

Building on promising pilot results, this paper presents the study protocol for an RCT of a group intervention for parents of children with a life threatening illness or injury, delivered using videoconferencing technology. Recruitment and intervention delivery for this trial are underway.

**Fig. 1:**
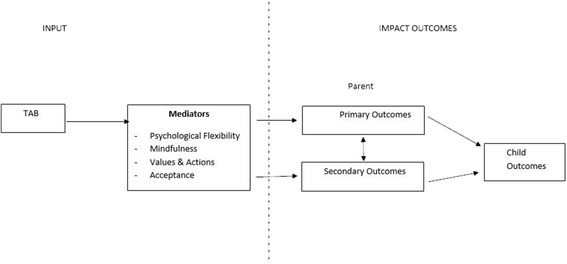
Take a Breath Program Logic Model

The aim of the trial is to determine the immediate and longer term efficacy of the TAB program in reducing parental distress, including posttraumatic stress symptoms (primary outcomes), in parents of children with life threatening illness or injury. As indicated in Fig. 1, we predict this reduction will arise from improvements in the psychological skills addressed in the intervention (mediating factors), and will be associated with broader improvements in parents’ adjustment (e.g., quality of life; and child adjustment and quality of life; secondary outcomes).

Specifically, we hypothesized that compared to waitlist control group, parents who receive the intervention will report the following short term (post intervention) outcomes:

- Fewer distress symptoms (primary outcome, assessed in terms of traumatic stress, general stress, anxiety and depression symptoms);
- Greater improvements in the psychological skills directly targeted by the intervention (mediating factors: e.g. improved psychological flexibility; mindfulness; reduced impact of their child’s illness);
- Improved parent adjustment (e.g., greater quality of life, improved condition management and parent mutuality), and
- Greater improvements in child adjustment and wellbeing.

We predict these short term benefits will be maintained in the longer term (6 month follow-up). Finally, we predict that within the intervention sample, there will be no differences in outcomes by child’s type of illness or age.

## Method

### Approval and registration

Ethics approval was granted by the Human Research Ethics Committee of the Royal Children’s Hospital (RCH), Melbourne (HREC 31049). The study is registered with the Australian New Zealand Clinical Trials Registry (ID: ACTRN12611000090910).

### Design

This superiority trial employs an RCT methodology in accord with CONSORT guidelines [34]. Parent-reported data are collected via questionnaires administered at four time points: T1 Screening within 4 weeks of the child’s admission/diagnosis to determine eligibility; T2 (Pre Intervention) completed 2 weeks prior to participating in the intervention (4–10 months after the child’s admission/diagnosis); T3 (Post Intervention) completed immediately after the intervention; and T4 follow up six months after completion of the intervention. Data are collected from waitlist and intervention participants at T1, T2 and T3, and from intervention participants only at T4.

### Setting

Participants are recruited from the Cardiology, Oncology, and Neurology Departments, and the Paediatric Intensive Care Unit (PICU) at the RCH, Melbourne, Australia, a statewide, tertiary paediatric hospital. These departments have been chosen for their high admission rates, the relatively sudden onset of the child’s illness, and the significant threat posed to the child’s development and future functioning. They also provide diverse illness groups that vary in child age at admission and the nature of treatment received.

### Participants

Participants are parents who are the primary caregiver of children aged 0–18 years. Parents are eligible if 1) their child is admitted for the first time for: cardiac surgery within the first month of life; a new cancer diagnosis; moderate or severe traumatic brain injury or stroke; or admitted to intensive care for more than 48 h, and 2) the parent reports elevated symptoms of acute distress as determined using the cluster cut-off score on the Acute Stress Disorder Scale (ASDS, see Table 1). Parents are required to be aged over 18 years and have sufficient English to participate. Exclusion criteria include pre-existing psychotic disorder, experience of another major trauma (e.g., death of family member) in the 2 months prior to child diagnosis and if the child is considered palliative. If, during the course of participation in the trial, eligible parents meet any of the exclusion criteria (e.g. their child passing away), they will be excluded from further participation. They will also be excluded if they suffer a serious or intolerable adverse event. Parents who voluntarily withdraw from the study will also be excluded.

**Table 1:** Parent-reported screening and outcome measures

Construct	Measure	Description	Cut points for inclusion in the trial	Timepoints administered^a^
Mediating factors Cognitive/psychological skills	Acceptance and Action Questionnaire – II (AAQ-II; [39]).	7 items assessing psychological flexibility/inflexibility (acceptance and experiential avoidance); e.g. *“I’m afraid of my feelings”* rated on a 7-point scale (1 = never true, 7 = always true). Higher scores indicate greater experiential avoidance and immobility; lower scores indicate greater action and acceptance. Mean Cronbach’s α across studies = .84.	n/a^b^	Pre, post, follow-up
	Parental Psychological Flexibility Questionnaire (PPF; [19]).	30 items assessing psychological flexibility (emotional willingness, cognitive defusion, acceptance) in relation to being a parent; e.g. *“My emotions get in the way of being the type of parent I would like to be”* rated on a 7-point scale (1 = never true, 7 = always true). Higher scores indicate poorer parental psychological flexibility. Cronbach’s α = .89 total scale, .90 cognitive fusion, .74 emotional willingness, .79 acceptance.	n/a^b^	Pre, post, follow-up
	Five Facet Mindfulness Questionnaire – Short Form (FFMQ-SF; [40])	24 items assessing general tendency towards day to day mindfulness. The five facets include: Observing, Describing, Acting, Nonjudging of Inner Experience, and Nonreactivity to Inner Experience. Items are rated on a 5-point scale (1 = never or very rarely true, 5 = very often or always true). For all facets, higher scores reflect higher levels of mindfulness. The five facets demonstrated adequate to good internal consistency, Cronbach’s α = .75–.87 [41]	n/a^b^	Pre, post, follow-up
	Valuing Questionnaire (VQ; [42])	8-items assessing the degree to which people live by their values. The VQ consist of 2 subscales: Progress (extent to which people felt that they lived their values in the past week) and Obstructed (the extent to which cognitive and emotional barriers interfered with acting out their values in the past week). Items are rated on 6-point scale (0 = not true at all, 6 = completely true). Cronbach’s α for the Progress and Obstructed subscales are .90 and .83, respectively.	n/a^b^	Pre, post, follow-up
Primary outcomes				
Parent Mental Health	Posttraumatic Stress Disorder Checklist – Specific (PCL-S; [36])	17 items assessing the symptoms of re-experiencing, avoidance and arousal over the past month; e.g. *“Feeling as if your future will somehow be cut short?*” rated on a 5-point scale (1 = not at all, 5 = extremely). Higher scores indicate greater symptoms of posttraumatic stress. Cronbach’s α = .97 total scale, .93 re-experiencing, .92 avoidance, and .92 arousal subscales [43].	n/a^b^	Pre, post, follow-up
	Depression Anxiety Stress Scale (DASS; [37])	21 items assessing symptoms of depression, anxiety, and stress or tension experienced over the past week; e.g. *“I couldn’t seem to experience any positive feeling at all”* rated on a 4-point scale (0 = does not apply to me at all, 3 = does apply to me very much or most of the time). Response are summed and multiplied by 2 to obtain a total score for each scale. Higher scores indicate greater symptoms. Cronbach’s α = .88 depression, .82 anxiety, and .90 stress [44].	Scores indicating mild or higher symptoms on any subscale: >5 for depression; >4 for anxiety; >8 for stress.	Pre, post, follow-up
Secondary outcomes				
Parent Adjustment	World Health Organization Quality of Life – BREF (WHOQol – BR EF; [45])	26 items assessing perceptions of ones quality of life; e.g. *“How much do you enjoy life”* The WHOQol consists of four domains of quality of life: physical health, psychological, social relations and environment. All items are rated on a 5-point scale (1 = an extreme amount, 5 = not at all). Cronbach’s α = .71–.85.	n/a^b^	Pre, post, follow-up
	Posttraumatic Growth Inventory – Short Form (PTGI; [46])	10 items assessing positive changes in individuals who have experienced highly challenging life circumstances; e.g. *“I have changed my priorities about what is important in life*” rated on a 5-point scale (0 = I did not experience this change as a result of my child’s illness/injury, 5 = I experienced this change to a very great degree as a result of my child’s illness/injury). Higher scores indicate increased positive changes since child’s diagnosis. Cronbach’s α = .86.	n/a^b^	Pre, post
	Parent Experience of Child Illness (PECI; [47])	25 items assessing parent adjustment (guilt and worry; emotional resources; unresolved sorrow and anger; long-term uncertainty) to a child’s serious or chronic illness; e.g. *“I worry that any minute, things might take a turn for the worse”* rated on a 5-point scale (0 = never, 4 = always). Higher scores indicate poorer adjustment to a child’s illness. Cronbach’s α = .72–.89.	n/a^b^	Pre, post, follow-up
	Family Management Measure (FaMM; [48])	Condition Management Ability Subscale 12 items assessing how families manage caring for a child with a chronic condition; e.g. “*It is often difficult to know when our child’s illness must come first in our family”* rated on a 5-point scale (0 = Strongly Disagree, 5 = Strongly Agree). Higher scores indicate increased positive changes since child’s diagnosis. Subscale Cronbach’s α = .73	n/a^b^	Pre, post, follow-up
Child Adjustment	Brief Infant-Toddler Social and Emotional Scale (BITSEA; [49])	42 items assessing parent perceptions of their infant or child’s (12–36 month olds) difficult behaviours and social-emotional problems which fall into seven domains: internalizing, externalizing, dysregulation, competence, social relatedness, maladaptive, and atypical (e.g. “Your child: Hits, bites or kicks you”). Items are rated on a 3-point scale (0 = not true/rarely, 2 = very true/often), with higher scores indicating greater levels of social-emotional or behavioural problems. The BITSEA has good internal consistency and inter-rater reliability, and excellent test-retest reliability [50]. Internalising, externalising and dysregulation subscales will only be administered.	n/a^b^	Pre, post, follow-up
*OR (depending on age of child)*				
	Behavior Assessment System for Children, Second Edition (BASC-2; [51])	134-items for children aged 2–5 years, and 160-items for children aged over 6 years which assess maladaptive and adaptive behaviours and self-perceptions of children. Items (e.g. ‘Is easily upset’; ‘Has trouble making new friends’), are rated on a 4-point scale (1 = never, 4 = almost always). There are nine clinical scales: Hyperactivity, Aggression, Conduct Problems, Anxiety, Depression, Somatization, Atypicality, Withdrawal, and Attention Problems; and three adaptive scales: Adaptability, Social Skills, and Leadership. There are also four composite scores: Externalizing Problems, Internalizing Problems, Behavioral Symptoms Index, and Adaptive Skills. Cronbach’s α range from mid .80s to mid .90s [52].	n/a^b^	Pre, post, follow-up
Child Wellbeing	Paediatrics Quality of Life [PedsQL; 53].	23 item assessing health-related quality of life in children and adolescents across four dimensions: Physical, Emotional, Social, and School functioning. Items (e.g. “In the past one month how much has your child had problems with feeling sad or blue”) are rated on a five-point Likert scale (1 = never a problem, 5 = always a problem). Higher scores indicate a better quality of life. Cronbach’s α for the parent report Total Scale Score = .90, Physical Health Summary Score.88, and Psychosocial Health Summary Score 0.86 parent) [53].	n/a^b^	Pre, post, follow-up
Screening, demographic and potential confounders				
Acute stress	Acute Stress Disorder Scale [ASDS: [54]).	19 items assessing acute stress disorder in individuals in the acute period (up to 4 weeks) following a traumatic event e.g. *“during the trauma did you ever feel numb or distant from your emotions?”* Measures 4 cluster of symptoms - dissociation (5 items), re-experiencing (4 items), avoidance (4 items) and arousal (6 items) rated on a 4-point scale (1 = Not at All to 5 = Very Much). Scores are summed. Internal consistency for the total scale was reported to be .96 and .84 for dissociation, .87 for experiencing, .92 for avoidance and .93 for arousal [54].	Scores of 9 or above on the first 5 items and 28 or above on the remaining 14 items.	Screening
Parent Demographic Factors	Demographics	Participant demographic information was collected, including age, gender, employment status, education attainment, relationship status, partners employment status, partner education attainment, country of birth, languages spoken, child’s age, child’s gender, number of other family members, service usage, and other significant life events.	n/a^b^	Screening
	Life Events Questions	The life events questionnaire was developed by the senior investigators to obtain information about potential psychosocial stressors that participating parents may have experienced in the past 12 months (in addition to their child’s serious illness/injury). The questionnaire contains a total of 14 items and asks about job loss and reduced work hours, recent pregnancies, moving home, suffering a serious illness/injury themselves, separation/divorce, or whether they have experienced an event they found traumatic. The parent is also asked to report on their partner. In addition to these items, a final 15^th^ item was included to ask about a history of mental illness in the year prior to their child’s illness/injury.	n/a^b^	Pre, post, follow-up
	Health Economic Questions	14 items assessing the potential impact of their child’s illness/disability on financial and employment conditions, as well as the health economic impact of the intervention. This measure is un-validated and was developed in consultation with a Health Economist and are commonly used by health economists to conduct an economic evaluation of the impact of the child’s illness/injury on the parents and family.	n/a^b^	
Intervention Acceptability	Consumer Satisfaction Scale [55].	19 items assessing consumer satisfaction with the quality of the service provided; how well the program met the parent’s needs and changed behaviour, and whether the parent would recommend the program to others. Parents are also prompted to make general comments or suggestions about the program.	n/a^b^	Post

^a^Screening = within 4 weeks of child’s initial hospital admission; pre = prior to intervention (4–6 months post admission); post = immediately after completion of the intervention; follow-up = 6 months after completion of the intervention. ^b^ *n/a* not applicable

### Recruitment and allocation

After consultation with the child’s medical team to determine potential eligibility, parents are approached on the hospital ward (or by phone if already discharged) by a member of the research team, to seek participation. Employing the support of consultants and clinical staff in the recruitment process will assist the research team to recruit as many eligible families as possible that pass through the relevant hospital departments, in order to achieve the adequate enrolment required to reach the target sample size. Written consent is obtained from eligible parents willing to participate, and subsequently are asked to complete the demographics and screening measure (ASDS) within four weeks of their child’s admission (T1). Eligible parents based on the screening measure are then phoned from four months post admission and invited to participate in the trial, with interested parents asked to sign a second written consent form. At this point they are asked to complete pre-intervention assessment measures (T2) and are randomly allocated to intervention or waitlist control group. The randomization list is generated independent of the research team by the hospital’s Clinical Epidemiology and Biostatistics Unit, using a computerized randomization plan generator and the method of randomly permuted blocks. The end result will be two randomly allocated treatment arms divided between four randomization lists, one for each department: Cardiology, Oncology, Neurology and PICU. The allocation sequence will be implemented using sequentially numbered, sealed envelopes, which will be controlled by an independent researcher who is not involved in recruiting families or intervention delivery. This will ensure that project staff are blind to client allocation. This independent researcher will also assign participants to the relevant treatment arm. At the time of completing their pre-intervention assessment, participants are blinded to allocation condition: they all have agreed to the assessments and program participation but are unaware of whether they are allocated to immediate or waitlist intervention.

Strategies will be employed to improve engagement and reduce attrition. These include giving parents keep cups, pens, and note pads with the study logo, to serve as reminders of study participation at home. Regular contact via phone or email with families throughout the trial will also be used to maximise engagement of families in the trial and reduce attrition during follow-up stages.

### Intervention delivery

TAB is a six-session ACT-based group program. It comprises five 90 min weekly sessions, with a sixth booster session scheduled three weeks after the fifth session. Parents participate from their home using a group videoconferencing platform on a study-provided tablet computer. Development and a feasibility trial of the online delivery format have been reported elsewhere [20]. Session content and structure were revised on the basis of the feasibility study and are summarized in Table 2. Participants are sent session materials designed to enhance the delivery of an online intervention, including a set of values cards, a session booklet, and a guided mindfulness CD and mp3. The intervention is co-facilitated by two experienced psychologists and mental health clinicians trained in the content and delivery of the program. Parents’ partners are invited to attend with a maximum group size of 8 parents/partners plus two facilitators.

**Table 2:** Session content and structure for the take a breath program

Session 1	Introductions
	Orientation to technology
	Group rules and process
	Overview of program and introduction to BOLD analogy
	Sharing stories
	“B” – Breathe deeply and slow down
	Mindfulness practice
	Wrap up and set home practice
Session 2	Mindfulness practice
	Home practice review
	“O” – Observing feelings
	Self-compassion
	Wrap up and set home practice
Session 3	Mindfulness practice
	Home practice review
	“O” – Observing thoughts
	Wrap up and set home practice
Session 4	Mindfulness practice
	Home practice review
	“L” – Listen to your values
	“D” – Decide what matters and do it
	Wrap up and set home practice
Session 5	Mindfulness practice
	Home practice review
	Group discussion and trouble shooting
	Recap of program overview
	Self as context
	Wrap up and set home practice
Break	
Session 6 (Booster)	Mindfulness practice
	Home practice review
	Group discussion and trouble shooting
	Functional behaviour change question
	Recap of program overview
	Wrap up and set home practice

#### Technology

Participants are sent iPads (16GB with Retina Display Wi-Fi + 3G) via registered mail to be used for the duration of the program. iPads are installed with the online videoconferencing platform Google Hangouts application [35], prepaid 3G data provided to allow for an internet connection, and accompanied by step-by-step user instructions. The GPS tracking function is enabled and the iPads are cased in a cover so the screen can stand on a table in landscape position. Technical checks are conducted over the phone with each participant in the week prior to program commencement, to test webcam and audio clarity and problem solve any issues. Group facilitators and participants share their webcam during sessions, so all screens display the weekly content materials as well as the faces and names of the facilitators and participants.

#### Facilitator training and program fidelity

Clinicians were trained in ACT with a trauma focus by RW, and were trained in the TAB intervention by MR prior to commencement of the trial. Group sessions are recorded and sections of a recording are reviewed fortnightly in group supervision sessions with RW and MR. The fidelity of program delivery across groups is measured using session monitoring checklists completed by both facilitators at the completion of each session. The checklist records content covered within each session, including any planned and omitted content, and measures group cohesiveness, active participation by group members, distractions and disruptions, quality of environment and physical resources, level of rapport and engagement, timing, technical problems and facilitators’ comfort and fluency with the material. Clinicians listen to a randomly chosen recording and score a fidelity monitoring checklist for one session of the groups where they are not facilitator or co-facilitator.

### Waitlist group

Families allocated to the waitlist group will receive standard clinical care within the hospital, including access to nurses, consultants, social workers, psychologists and other allied health clinicians as required. Families within the treatment group will also have access to standard care.

### Measures

Measures and data collection time-points are summarized in Table 1. The primary outcome of parent mental health is assessed using parent-report measures of posttraumatic stress symptoms, depression, anxiety, and general stress, using the Posttraumatic Stress Disorder Checklist – Specific (PCL-S) [36] and the Depression Anxiety Stress Scale (DASS) [37] respectively. Other measures assess the hypothesized mediating factors addressed by the intervention (e.g. parent valued living, mindfulness, experience of child’s illness). Secondary outcomes of parent adjustment (e.g. quality of life, management of the condition) and child adjustment (e.g. social, emotional and behavior difficulties) and child wellbeing are also assessed. All are assessed at T2-T4, using reliable and validated parent-report measures. Parent demographic factors (e.g. age, gender, income, ethnicity, education) are collected at screening or T2 and intervention parents complete an intervention acceptability measure at T3. Details of the child’s illness (e.g. diagnosis, date of diagnosis, number of visits to the Emergency Department and number of days of admission) are extracted from hospital databases.

### Participant flow and estimated sample size

**Fig. 2:**
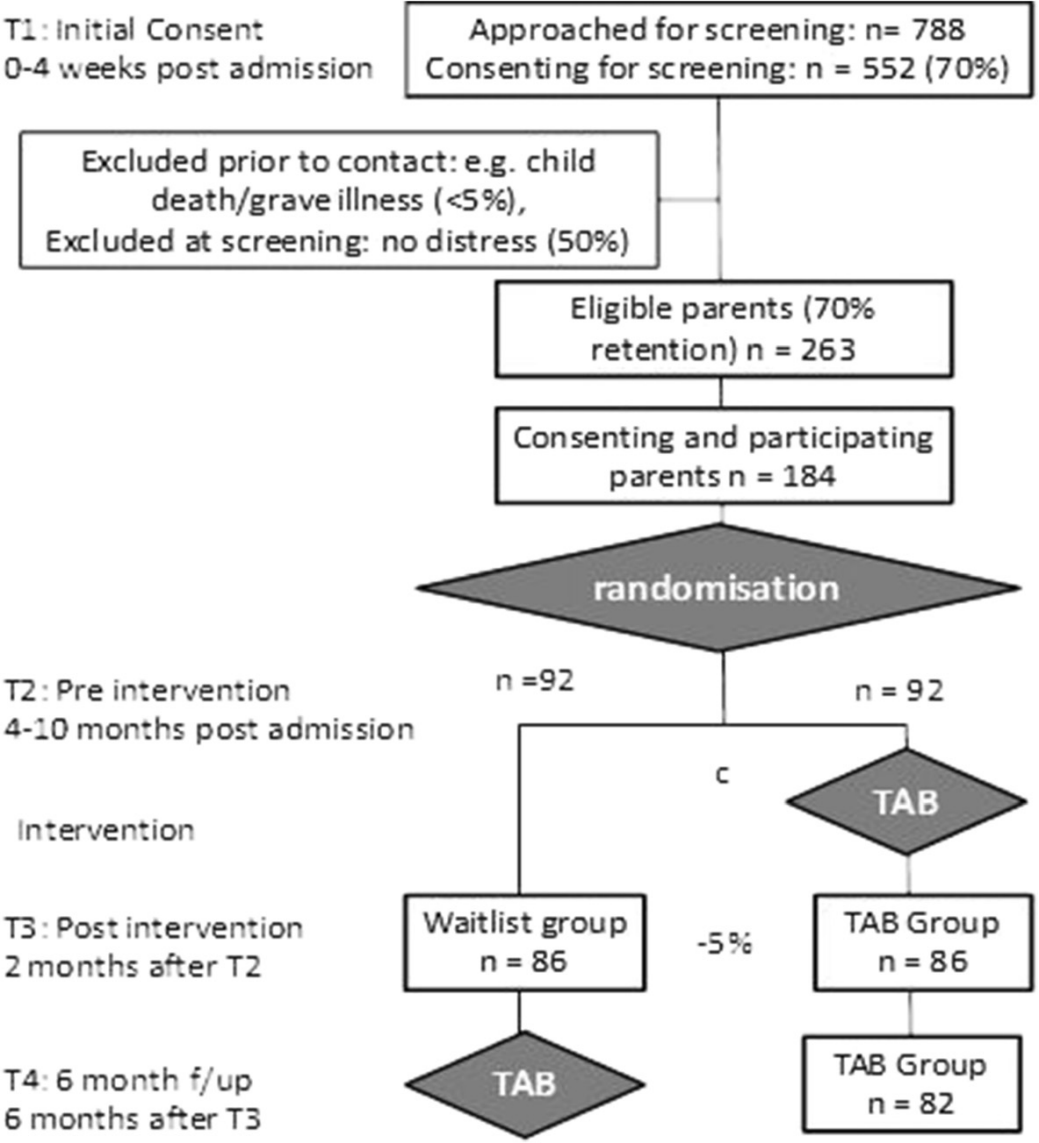
Recruitment, assessment & participant flow

Figure 2 outlines the anticipated participant flow based our previous research experience with this population and the required numbers to obtain sufficient power. In order to detect a clinically meaningful difference between the two treatment arms of 6.0 (SD: 13.9) points on the primary outcome (PCL-S), with significance level of 0.05 and power of 0.80, 86 participants per condition are required at the post intervention time point (T3). Allowing for the attrition indicated in Fig. 2, this requires a total *N* = 184 recruited and randomized.

### Data storage

The source data will be managed using a RedCAP database which will be held at on campus in a secure room on a password protected computer. Hard copy consent forms and questionnaires will be kept separately in a locked compactus on campus. All audio files recorded during intervention delivery will be stored on a password and firewall protected computer in a locked room. In order to maintain security and confidentiality, only members of the research team with approval from the Royal Children’s Hospital Ethics Committee will have access to any of the personal information and data collected from all potential and participating participants, both throughout and after the conclusion of the trial.

### Data analysis

Primary analyses will be conducted following the intention-to-treat paradigm with all participants analyzed as allocated, irrespective of attendance. Analyses will use a generalized estimating equations approach to permit use of all available data. Any baseline imbalances that occur despite randomization will be accounted for in multivariable linear and logistic regression modelling. Likely confounders and moderators (e.g. partner participation or not; child’s diagnostic group, illness severity and age; parent and family characteristics) will be explored. Supplementary analyses will include per-protocol analyses and accounting for attendance and retention bias by comparing the sensitivity of conclusions to various strategies for managing or imputing missing data.

### Trial management

The project will be coordinated and overseen by a chief investigator team (MR, FM, MM, VA, JN), who will communicate any important protocol deviations to the remaining project investigators, the Royal Children’s Hospital Ethics Committee, the trial registry, and to relevant journals. They will also be responsible for assessing, reporting and managing adverse events throughout the trial. Any serious adverse events will be recorded and reported to the Royal Children’s Hospital Ethics committee within 72 h of occurrence. Any parent who suffers from severe distress within the program, or as a result of the program, will be supported by the team to engage professional mental health services either within the hospital or in the community, as appropriate.

The chief investigator team will meet monthly to discuss study procedures, problems that arise, and monitor the conduct of the trial, and adherence to the protocol. The two most senior members of the chief investigator team (VA and JN) will comprise the data monitoring committee. They will oversee any interim analyses should they be required throughout the trial, and will determine if participant recruitment will be able to be ceased at an earlier stage. This committee, and any analyses they conduct will remain independent and confidential to the remainder of the team.

### Strengths and limitations

This study has notable design strengths: inclusion of parents of children across a diverse range of life threatening conditions, who have elevated symptoms of acute stress at the time of recruitment; timing the intervention to avoid the acute period when parents face intense demands around their child’s medical care; a group delivery format that is more cost-efficient than individual therapy and enables normalization of experience and peer support; use of videoconferencing, paired with clinician input to reduce participation barriers and maximize effectiveness; and the first use of an ACT-based approach in this population. CONSORT guidelines [34] have been followed to maximise study validity.

The major design weakness is the use of the waitlist control condition with waitlisted parents offered the program after completing the ‘post’ (T3) assessment. This precludes comparison between intervention and control participants over the longer term (i.e., at 6 month follow-up). While not ideal, this design reflects the realities of ethical research practice with this highly vulnerable group. Our research team has been conducting research with this population for over 5 years, and it is both practically and ethically challenging to undertake repeated assessments of distressed participants over a long time periods without providing any support. High rates of drop-out by non-intervention participants makes the representativeness of the data questionable for those who are retained, and options for imputing missing data are limited by high rates and non-randomness of the losses to follow-up. Our team has collected longitudinal data from a large cohort of parents of children with life-threatening illnesses recruited in the same setting and using similar eligibility criteria, measurement tools, and assessment time points [38]. The large sample size for this observational study (145 parents assessed at the equivalent of the current study’s proposed 6 month follow-up) provides us the opportunity to compare the long term adjustment of intervention families from the current study with a non-intervention normative population.

## Discussion

One in five parents whose child is diagnosed with a life threatening condition experiences enduring and debilitating distress that adversely affects daily functioning and the wellbeing of other family members [14]. Importantly, addressing distress associated with medical illness has become a central tenet of modern health service delivery. For example, in oncology, distress as the 6th vital sign has been endorsed by numerous policy and organisational bodies [1]. As a result, screening for distress has been mandated as a quality standard in the USA, required for hospital accreditation. There are, however, no effective, evidence-based interventions to address distress in patients and families, once identified. If effective, this program will be an important step to addressing this need and will address a serious gap in the provision of evidence-based care within the child healthcare system. Specifically, it has the potential to reduce burden within families, improve the quality of life of parents, the ill child and other children in the family, thereby reducing demand on hospital and community services.

In addition, advances in communication technology, combined with near-universal connectivity of the population to online and mobile devices, is radically changing healthcare delivery. This study will build our understanding of how such technology can be used to provide widespread and cost effective access to expert clinicians delivering specialist psychological interventions. Importantly, the online format reduces established mental health service inequalities for those in rural/remote locations, and addresses the reluctance of struggling parents to leave their ill child in the care of others, in order to obtain needed support. The use of a videoconferencing group platform provides the additional advantage of enabling both parents to participate in the intervention together from home, sharing the experience and learning together. This is a rare opportunity for families engaged in interventions.

A key feature of TAB is the use of a trans-diagnostic therapeutic model (ACT) and its applicability across multiple diagnostic groups. ACT focuses on acceptance of painful emotions and difficult thoughts while continuing to behave in values-consistent ways. This approach may assist parents in managing challenging internal experiences in the midst of an overwhelming and, at times, quite frightening event while still focusing on what matters most in the family and care-giving environment. If its effectiveness is supported in the current trial, this approach has the potential to be implemented as a hospital-wide program.

## Abbreviations

ACT: acceptance and commitment therapy
ASDS: acute stress disorder scale
DASS: depression anxiety stress scale
PCL-S: posttraumatic stress disorder checklist – specific trauma version
PICU: pediatric intensive care unit
RCH: Royal Children’s Hospital
RCT: randomized controlled trial
T1-T4: data collection time points 1 to 4
TAB: take a breath
